# Role of Interleukin-34 in Cancer

**DOI:** 10.3390/cancers12010252

**Published:** 2020-01-20

**Authors:** Eleonora Franzè, Carmine Stolfi, Edoardo Troncone, Patrizio Scarozza, Giovanni Monteleone

**Affiliations:** Department of Systems Medicine, University of Rome “TOR VERGATA”, 00133 Rome, Italy; eleonorafranze@yahoo.it (E.F.); carmine.stolfi@uniroma2.it (C.S.); troncone.edoardo@gmail.com (E.T.); scarozzapatrizio@gmail.com (P.S.)

**Keywords:** interleukins, tumor microenvironment, tumor associated macrophages, M-CSF1-R

## Abstract

Cross-talk between cancer cells and the immune cells occurring in the tumor microenvironment is crucial in promoting signals that foster tumor growth and metastasis. Both cancer cells and immune cells secrete various interleukins (IL), which, either directly or indirectly, stimulate cancer-cell proliferation, survival, and diffusion, as well as contribute to sculpt the immune microenvironment, thereby amplifying tumorigenic stimuli. IL-34, a cytokine produced by a wide range of cells, has been initially involved in the control of differentiation, proliferation, and survival of myeloid cells. More recent studies documented the overexpression of IL-34 in several cancers, such as hepatocarcinoma, osteosarcoma, multiple myeloma, colon cancer, and lung cancer, and showed that tumor cells can produce and functionally respond to this cytokine. In this review, we summarize the multiple roles of IL-34 in various cancers, with the aim to better understand the relationship between the expression of this cytokine and cancer behavior and to provide new insights for exploring a new potential therapeutic target.

## 1. Introduction

Cancer development relies on multiple genetic alterations which favor progression of transformed cells to a malignant condition and several components of the tumor microenvironment, including both immune and nonimmune cells and extracellular matrix [1]. Indeed, cancer cells have the ability to recruit and activate non-transformed fibroblasts, endothelial cells, and many subsets of immune cells, with the downstream effect of generating a microenvironment favorable to disease progression [2]. Active and dynamic cross-talk between these components of the tumor microenvironment and the neoplastic cells is crucial in providing signals that enhance cancer cell growth and survival, as well as the metastatic property of tumor cells [3]. The tumor–stroma interaction can occur both directly, via cell–cell interaction/communication, and through the action of a bunch of molecules secreted by both neoplastic cells and cancer-associated immune/nonimmune cells [4]. Identification of such molecules has largely advanced our understanding of the mechanisms promoting cancer initiation and progression and facilitated the development of drugs useful to combat cancer.

In this article, we summarize the multiple roles of IL-34 in various cancers, with the aim to better clarify the relationship between the expression of this cytokine and cancer behavior and to provide new insights for exploring a new potential therapeutic target.

## 2. Interleukin-34

The human IL-34 was identified by Lin and colleagues in 2008 (named in a public database C16orf77) as a selective protein that promotes monocyte viability [5]. The *IL-34* gene is located on chromosome 16q22.1, whereas the mouse ortholog (i.e., *Il-34*) maps to chromosome 8E1. Human IL-34 shares an amino acid sequence identity of 99.6%, 72%, and 71% with IL-34 of the chimpanzee, rat, and mouse, respectively [5]. IL-34 shows no supposed consensus structural domain/motif, nor a sequence similarity with any other growth factor, including macrophage colony-stimulating factor (M-CSF-1; also known as CSF-1) [6]. The full-length mature human IL-34 protein is composed of 242 amino acids (235 amino acids in mouse), with a molecular mass of 39 KDa. The first 182 amino acids contain predicted N-glycosylation sites at Asn76 and Asn100 positions, which are crucial for IL-34 stability and correct folding, and six cysteine residues that are highly maintained among species [5,6,7]. Although IL-34 has no sequence homology with M-CSF-1, it exerts its biological function through the interaction with the homodimeric M-CSF-1 receptor (M-CSF1-R; also known as CSF1-R, CD115, FMS) [5,8]. Crystallographic experiments showed that the non-covalently linked IL-34 homodimer recruits 2 copies of M-CSF1-R on the sides of the helix bundles. IL-34 binds to a concave surface made by the N-terminal immunoglobulin D2 and D3 domains of M-CSF1-R, whereas the D4 domain is likely involved in the IL-34-induced oligomerization [6,7,8,9]. Despite IL-34 and M-CSF-1 share the same receptor, the two cytokines can activate different signaling pathways and mediate distinct biological functions [10]. This relies in part on the different hydrophobic/hydrophilic interactions of each cytokine with M-CSF1-R. Conversely to the M-CSF-1:M-CSF1-R complex, which depends on hydrophilic interactions, the IL-34:M-CSF1-R interface bears several hydrophobic regions, which appear to be relevant for stabilizing the cytokine-receptor binding and favoring a prolonged and strong transmembrane signaling [5]. It has also been shown that IL-34 and M-CSF-1 bind different anchorage points of M-CSF1-R, thereby triggering distinct signaling pathways [11].

**Figure 1:**
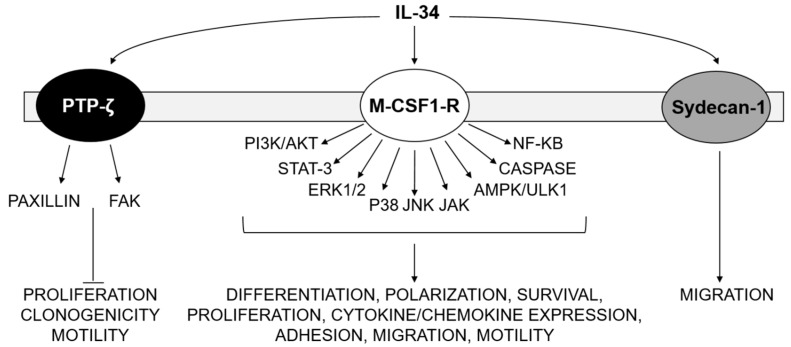
IL-34-driven signaling pathways. IL-34 binds to M-CSF1-R, PTP-ζ and Sydecan-1, activating several signaling pathways that regulate major cellular functions, including differentiation, polarization, survival, proliferation cytokine/chemokine expression, motility, and migration.

Depending on the cell type and context analyzed, binding of IL-34 to M-CSF1-R triggers different signaling pathways, such as NF-κB, phosphoinositide 3-kinase (PI3K)/AKT, p38 mitogen-activated protein kinase (MAPK), extracellular signal-regulated protein kinases 1 and 2 (ERK1/2), c-Jun N-terminal kinase (JNK), Janus kinase (JAK), signal transducer, and activator of transcription (STAT)3 [9,10,12,13,14] (Figure 1). IL-34-induced signals can also activate caspase-3/8 and promote autophagy through an AMP-activated protein kinase-UNC-51-like Kinase 1-dependent mechanism [15] (Figure 1). By investigating the expression pattern of IL-34 in the brain, Nandi and colleagues documented the presence of the cytokine in areas where there was a minimal expression of M-CSF1-R, raising the possibility that IL-34 could signal via an alternative receptor [16]. Indeed, it is now known that the receptor-type protein–tyrosine phosphatase zeta (PTP-ζ) a cell surface chondroitin sulfate proteoglycan primarily expressed on neuronal progenitors and glial cells, and to lesser extent on B cells and kidney tubular cells [16] (Figure 1). The interaction between IL-34 and PTP-ζ can induce a series of intracellular events that inhibit motility, clonogenicity, and proliferation of specific cell types via tyrosine phosphorylation of paxillin and focal adhesion kinase (FAK) [16] (Figure 1). More recently, Segaliny and collaborators identified Syndecan-1 (also known as CD138) as an additional functional IL-34 receptor, which, once engaged, stimulates myeloid cell migration [17] (Figure 1).

IL-34 is produced by different cell populations, including endothelial cells, adipocytes, neurons, macrophages, fibroblasts, and epithelial cells, and is constitutively expressed in several human tissues (e.g., brain, thymus, heart, liver, spleen, testis, prostate, ovary, small intestine, colon) [18,19,20,21]. There is also evidence that IL-34 production can change under pathological conditions [22]. Indeed, deregulated IL-34 expression has been documented in various immune-inflammatory disorders, infections, and metabolic and neurologic diseases [23]. Several factors can regulate IL-34 production. For instance, pro-inflammatory cytokines, such as TNF-α and IL-1β, enhance IL-34 synthesis in fibroblasts, epithelial cells, intestinal lamina propria mononuclear cells (LPMC), periodontal ligament cells, osteosarcoma cells, and adipocytes, through NF-κB- and MAP kinase-dependent pathways [13,24,25,26]. Moreover, activation of toll-like receptors (TLRs) with pathogen-associated molecular patterns, such as peptidoglycan, lipopolysaccharide, and nucleic acid mimickers (e.g., CpG, poly I:C) promotes IL-34 expression in macrophages and intestinal LPMC, as well as in adipocytes, while stimulation with Iα,25(OH)2D3, a hormonally active form of vitamin D, increases IL-34 expression in neuroblastoma cells and normal gastric epithelial cells [24,26,27]. Hepatitis C virus (HCV)-infected hepatocytes synthesize high levels of IL-34, suggesting that viruses might directly regulate IL-34 expression [21]. On the other hand, TGF-β1 and bone morphogenetic protein-2 inhibit IL-34 synthesis in TNF-α-stimulated synovial fibroblasts and mesenchymal stem cells [28].

IL-34 is detectable at low concentrations in saliva, serum, plasma, and synovial fluid [29,30,31]. A correlation has also been reported between changes in the content of IL-34 into these biological fluids and disease parameters in various pathological conditions, such as rheumatoid arthritis (RA), systemic lupus erythematosus, heart failure, viral infections, sepsis, periodontal disease, nonalcoholic fatty liver disease, obesity, and type 2 diabetes mellitus [32]. Several studies have shown that IL-34 level is a useful biomarker for predicting disease progression and responsiveness to biologics (i.e., TNF blockers) in RA [33].

### 2.1. IL-34 Expression in Cancer

High expression of IL-34 has been documented in various cancers, where the cytokine is supposed to play important roles in multiple aspects of the tumorigenesis. For instance, IL-34 expression was seen in giant cell tumors of bone [18] human osteosarcoma and human osteosarcoma cell lines, and it is associated with the progression of the neoplasia (that is, increased tumor growth and lung metastases), as well as with an increase of neo-angiogenesis [34]. A more pronounced expression of IL-34, together with high levels of M-CSF-1, was also seen in primary lung cancer tissues compared with normal lung tissues, where the elevated levels of the cytokine significantly correlate with poor prognosis [35]. Such observations suggest that IL-34 expression may serve as an important prognostic biomarker in lung cancer patients [35]. Moreover, as the expression of both IL-34 and M-CSF1-R in primary lung adenocarcinoma cells increases upon exposure to doxorubicin or cisplatin, IL-34 protein levels may also serve as a biomarker to monitor chemoresistance insurgence and progression in cancer patients receiving chemotherapy [36]. The contribution of IL-34 in cancer is also supported by many other studies (Table 1), documenting an enhanced expression of the cytokine in various neoplastic diseases, including hematological malignancies, brain, breast, neck, biliary, and ovarian cancer [37,38]. Consistent with a pro-tumorigenic role of IL-34, many studies have documented the expression of the functional receptors of IL-34 in a variety of cancers [32].

### 2.2. Involvement of IL-34 in Cancer Initiation and Progression

**Figure 2:**
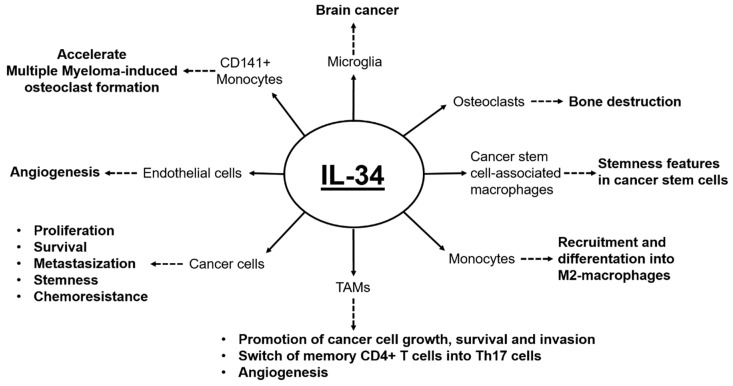
Overview of the tumor promoting effects of IL-34 on its target cells. Solid arrows indicate the targets of IL-34. Dashed arrows indicate the IL-34-driven, target-related tumorigenic effects.

Accumulating evidence suggests that IL-34 acts through autocrine and paracrine mechanisms to promote carcinogenesis (Figure 2).

In the autocrine pathway, IL-34 interacts with the M-CSF1-R on cancer cells, thereby activating signaling pathways that stimulate cancer cell growth and diffusion and/or enhance their resistance to chemotherapeutic drugs [36]. In the paracrine pathway, IL-34 produced by neoplastic cells and/or immune cells triggers the M-CSF1-R signaling in tumor-associated macrophages (TAMs), thus promoting their recruitment to the tumor area, favors the formation of new vessels, as well as the extravasation of immune-inflammatory cells [54]. These later observations are consistent with the demonstration that M-CSF1-R-driven signals are required for the survival and function of TAMs [55]. In the tumor microenvironment, IL-34 affects TAM functions in different ways. In mice, embryonic stem cell (ESC)-derived IL-34 contributes to polarizing bone marrow-derived macrophages into M2-like macrophages, which exhibit TAM phenotypic and functional features (e.g., increased levels of Arg-1, Tie-2, TNF-α) and contribute to angiogenesis and teratoma progression [44]. Depletion of macrophages completely inhibits ESC-induced angiogenesis and teratoma development. Moreover, IL-34 promotes macrophage survival via ERK1/2 and PI3K pathways [44].

In lung cancers, IL-34 triggers activation of CCAAT/enhancer-binding protein β via AKT-mediated pathway with the downstream effect of enhancing the pro-tumorigenic and immunosuppressive functions of TAMs and consequently promoting the survival of chemoresistant cancer cells [36]. IL-34 is also expressed in human ovarian cancer cell lines and tissues [43]. IL-34 levels increase in ovarian cancer cell lines following treatment with cytotoxic agents and in the tumor tissues of chemotherapy-given ovarian cancer patients [43]. Notably, high IL-34 expression correlates with worse progression-free survival and overall survival in different cohorts and in a mouse model of ovarian cancer [43]. Moreover, Han et al. reported enhanced expression of IL-34 in a patient with metastatic melanoma who acquired resistance to anti-PD-1 immunotherapy (Nivolumab) [53]. Cytotoxic therapies induce mammary epithelial cells to produce monocyte/macrophage recruitment factors, including M-CSF-1 and IL-34, which together enhance M-CSF1-R-dependent macrophage infiltration [39]. Blockade of macrophage recruitment through antagonists of the M-CSF1-R-signaling reduce primary tumor development and vessel density, promote antitumor immune programs, and decrease pulmonary metastasis, thereby improving survival of mammary tumor–bearing mice [39].

IL-34 also mediates the interaction between tumor cells and TAMs. In hepatocellular carcinoma (HCC), neoplastic cell-derived IL-34 stimulates TAMs to produce TGF-β1 [45]. TGF-β1 increases IL-34 synthesis in HCC cells by decreasing microRNA (miR)-28-5p, one of the miRNAs that dampen IL-34 production in HCC cells [45]. Notably, in patient-derived HCC specimens, IL-34 expression is inversely correlated with miR-28-5p levels and the presence of TAMs. HCC patients showing high IL-34 levels, low miR-28-5p expression, and an elevated number of TAMs had a poor prognosis with shorter overall survival and time to recurrence [45]. Moreover, IL-34 seems to educate TAMs to shape tumor-supportive immune niche [45]. Indeed, in cholangiocarcinoma, IL-34 produced by cancer stem cells (CSCs) stimulates macrophage infiltration, differentiation, and activation toward acquisition of CSC-associated macrophage phenotype [47]. IL-34 produced by giant cells of bone tumors supports RANKL-driven osteoclastogenesis, even in the absence M-CSF-1, by inducing M-CSF1-R-dependent activation of ERK 1/2 and Akt [18]. Moreover, in multiple myeloma (MM), IL-34 accelerates MM-induced osteoclast formation from monocytes and increases the severity of bone lesions [50], whereas, in sporadic vestibular schwannoma, IL-34 expression is not related to clinicopathologic characteristics and tumor growth [51]. Finally, in adult T-cell leukemia/lymphoma (ATLL), IL-34 and M-CSF-1 co-expression was found to correlate with tumor aggressiveness [52]. In this context, experimental studies using ATLL cell lines revealed that engagement of M-CSF1-R with either M-CSF-1 or IL-34 increased cell proliferation, thus suggesting a possible synergistic pro-tumorigenic effect of these two cytokines [52].

### 2.3. Counter-Regulatory Role of IL-34 in Cancer

**Figure 3:**
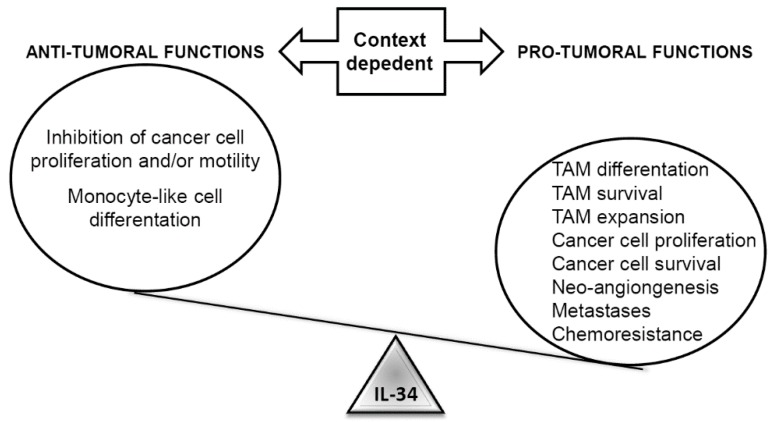
Schematic overview depicting the pro- and anti-tumorigenic functions of IL-34. IL-34 can exert both tumor and antitumor functions, depending on the tissue context. In some tumors, IL-34 inhibits cancer cell proliferation and/or motility, as well as monocyte-like cell differentiation. Conversely, in the majority of cases, IL-34 plays a tumor-promoting role by acting directly on transformed cells, to increase their proliferation/survival, indirectly favoring tumor-associated macrophage (TAM) differentiation, survival, and expansion.

In addition to the pro-tumorigenic activities indicated above, IL-34 plays unique roles in specific settings. For instance, IL-34 hampers proliferation, clonogenicity, and motility of glioblastoma cells and promotes the differentiation of monoblastic leukemia cells into monocyte-like cells [16,56]. Analysis of IL-34 RNA expression and breast cancer subtypes showed that high IL-34 expression correlated with a better prognosis in luminal and HER2 subtypes and worse prognosis in the basal one [41]. These findings were unexpected and surprising, as expression of both M-CSF-1 and M-CSF1-R has been linked to aggressive behavior and poor prognosis in breast cancer patients [57,58]. Analysis of the immune cells infiltrating the tumor also revealed a negative correlation between IL-34 expression and the immunosuppressive type-2 macrophages in luminal subtypes, whereas no correlation was reported in HER2 and basal subtypes [41]. In vitro experiments showed that IL-34 and M-CSF-1 differently modulate breast cancer cell migration, depending on the molecular subtype analyzed. In particular, IL-34 promotes cancer cell migration in basal breast cancer cells through a mechanism that is independent of M-CSF1-R, while it inhibits cancer cell migration in HER2-positive cells. In contrast, IL-34 has no effect on the migration of luminal cancer cells, which is regulated by M-CSF-1/M-CSF1-R [41]. Altogether, these data indicate that the role of IL-34 in cancers is extremely complex and likely context-dependent (Figure 3). 

### 2.4. Role of IL-34 in Colon Cancer

Colorectal carcinoma (CRC) is one of the most common forms of malignancy in the Western world. Circumstantial evidence suggests that colon carcinogenesis is tightly controlled by tumor-associated immune cells, which can either stimulate or suppress CRC cell growth and survival, mainly via the production of cytokines [59]. By using different molecular techniques, we have recently shown that tumor areas of patients with sporadic CRC contain elevated levels of IL-34 and M-CSF1-R compared to nontumor areas of the same CRC patients and normal controls [48]. By immunohistochemistry, we also reported that IL-34 was essentially produced by cancer cells and, to a lesser extent, by CRC-associated immune cells [48]. In line with previous studies performed in other systems, our work also showed that inflammatory cytokines overproduced in CRC tissue (i.e., IL-6, IL-17A, TNF-α, and IFN-γ) stimulated CRC cell lines to secrete IL-34. IL-34 enhanced CRC cell growth and invasion without affecting CRC cell survival. The proliferative effect of IL-34 occurred via an ERK1/2-dependent pathway. Consistently, IL-34 downregulation with a specific antisense oligonucleotide reduced activation of ERK1/2 and ELK1, a downstream target of ERK1/2, inhibited CRC cell growth, and enhanced the susceptibility of CRC cells to oxaliplatin-induced death [48]. More recently, Kobayashi et al. suggested that IL-34 may serve as a prognostic marker in CRC [49]. In this study, the authors demonstrated that IL-34 is detectable in various CRC cell lines, as well as in primary CRC tissues taken from a cohort of 292 Japanese patients. High expression of IL-34 correlated with an unfavorable prognosis and poor survival [49]. A similar correlation was also seen in a cohort of CRC patients registered at The Cancer Genome Atlas [49]. The authors also reported a positive correlation between IL-34 and the TAM-related marker CD163, whose expression associates with poor survival in CRC patients [49].

## 3. Conclusions

Since its discovery, IL-34 has received research attention for its ability to regulate a variety of cellular processes, including differentiation, proliferation, adhesion, metabolism, angiogenesis, inflammation, and immune responses. Moreover, it is becoming evident that interactions between IL-34 and its functional receptors trigger several intracellular pathways that ultimately control the growth and progression of many cancer types. In the majority of cancers, increased synthesis of IL-34 results in enhanced tumorigenesis, whereas, in few and specific settings, IL-34 can negatively control cancer cell growth and diffusion. However, further works are warranted to better understand the favorable and deleterious effects of IL-34 in the tumor microenvironment, as well as to ascertain whether IL-34 is a valid biomarker for the diagnosis and management of cancer. The factors and molecular mechanisms that regulate IL-34 in cancer cells remain unknown. It is conceivable that induction of IL-34 is linked to oncogenic mutations, as these have been reported to trigger signals leading to the production of cytokines and chemokines that sustain carcinogenesis [60]. Another possibility is that altered synthesis of IL-34 relies on changes in the expression of miRNAs, a class of small noncoding RNAs that regulate comprehensive biological processes by changing the expression and translation of their target messenger RNA genes [61]. Indeed, as stated above, IL-34 is a direct target of miR-28-5p in HCC metastasis [45]. Finally, molecules (e.g., cytokines) released within the tumor microenvironment by both immune and stromal cells may stimulate cancer cells to make IL-34. This is supported by the demonstration that IL-34 can be induced in cells exposed to inflammatory/tumorigenic cytokines (e.g., TNF-α, IL-1β, and IL-6) [24,50].

## Figures and Tables

**Table 1:** IL-34 expression in different cancer types.

Cancer Type	Functions	References
**Human bone giant cell tumor**	IL-34 is expressed in giant cell tumors and promotes RANKL-induced osteoclastogenesis	[18]
**Human/mouse mammary cancer**	Cytotoxic therapies induce mammary epithelial cells to produce monocyte/macrophage recruitment factors, including M-CSF-1 and IL-34, which together enhance M-CSF1-R–dependent macrophage infiltration. Blockade of macrophage recruitment with M-CSF1-R-signaling antagonists improves survival of mammary-tumor–bearing mice by slowing primary tumor development, reducing pulmonary metastasis, decreasing vessel density and appearance of antitumor immune programs, fostering tumor suppression in a CD8^+^T-cell–dependent manner	[39]
	Lung and brain metastases derived from breast cancer express M-CSF-1 and IL-34	[39]
	The IL-34 receptor Sydecan-1 (CD138) is increased in breast cancer and associates with high-grade tumors	[40]
	Expression of IL-34 is associated with a favorable prognosis in luminal and HER2, but not basal, breast cancer patients	[41]
**Human ovarian cancer**	IL-34-treated macrophages and TAMs switch memory but not naive CD4+ T cells into conventional Th17 cells, expressing or not IFN-γ via membrane IL-1α	[42]
	IL-34 is expressed in several human ovarian cancer cell lines and human ovarian cancer tissues. IL-34 expression increases following incubation of ovarian cancer cell lines with chemotherapeutic agents, as well as in the tumor tissues of chemotherapy-treated ovarian cancer patients. High IL-34 expression correlates with worse progression-free survival (PFS) and overall survival in different cohorts and in a mouse model of ovarian cancer	[43]
**Mouse teratoma**	Embryonic stem cells produce IL-34 that increases M2 macrophages and neo-angiogenesis	[44]
**Human hepatocellular carcinoma**	IL-34 is produced by hepatocellular carcinoma (HCC) cells, and it is able to enhance TGF-β expression in TAMs. TAM-derived TGF-β increases IL-34 production in HCC cells by decreasing miR-28-5p gene expression. The resulting IL-34-TAMs-TGF-β positive feedback loop mediates HCC cell growth and metastasis	[45]
	High IL-34 serum levels associate with poor prognosis in patients with non-viral HCC. IL-34 serum level is a key prognostic factor for patients with non-viral HCC	[46]
**Human/mouse osteosarcoma**	IL-34 is produced by osteosarcoma cells and its expression regulated by TNF-α and IL-1β. IL-34 enhances osteosarcoma growth and metastasization by increasing M2 macrophages recruitment and neo-angiogenesis	[34]
**Human cholangiocarcinoma**	IL-34 is produced by cancer stem cells (CSCs) and, together with other factors, induces macrophage infiltration, differentiation, and activation toward a CSC-associated macrophage phenotype, thus promoting stemness features in CSCs	[47]
**Human colorectal carcinoma**	IL-34 and its receptors M-CSFR-1 and PTP-ζ are highly expressed in colorectal carcinoma (CRC). IL-34 induces CRC cell proliferation and invasion through an ERK1/2-dependent mechanism. IL-34 knockdown reduces cell proliferation and enhances the susceptibility of CRC cells to oxaliplatin-induced death	[48]
	IL-34 is a prognostic factor in CRC and high IL-34 levels correlate with unfavorable prognosis in CRC patients	[49]
**Human lung cancer**	IL-34 and M-CSF-1 expression correlates with advanced tumor stages and poor survival in a cohort of lung cancer patients	[35]
	IL-34 triggers activation of CCAAT/enhancer-binding protein β via an AKT-mediated pathway. This enhances the tumorigenic and immunosuppressive functions of TAMs with the ultimate result to promote the survival of chemoresistant cancer cells	[36]
**Human/mouse multiple myeloma**	IL-34 expression is detected in multiple myeloma (MM) cells. IL-34 may have a pathological role in accelerating MM-induced osteoclast formation, both from human CD141 monocytes and mouse bone marrow cells, thus increasing the severity of bone lesions	[50]
	Targeting IL-34 by specific small interfering RNA impairs osteoclast formation in vitro and attenuated osteolytic disease in vivo	
**Human sporadic vestibular schwannoma**	IL-34 is expressed in sporadic vestibular schwannoma	[51]
**Human adult T-cell leukemia/lymphoma**	IL-34 is expressed in adult T-cell leukemia/lymphoma (ATLL). IL-34 co-expression with M-CSF-1 may be related to the aggressiveness of this cancer type	[52]
**Human refractory melanoma**	Enhanced expression of IL-34 in refractory melanoma tissues in a patient with metastatic refractory melanoma that acquired resistance to anti-PD-1 immunotherapy (Nivolumab)	[53]

TAMs = tumor associated macrophages.
